# Contrast, contours and the confusion effect in dazzle camouflage

**DOI:** 10.1098/rsos.160180

**Published:** 2016-07-06

**Authors:** Benedict G. Hogan, Nicholas E. Scott-Samuel, Innes C. Cuthill

**Affiliations:** 1 School of Biological Sciences, University of Bristol, Bristol, UK; 2 School of Experimental Psychology, University of Bristol, Bristol, UK

**Keywords:** confusion effect, dazzle camouflage, defensive coloration, target tracking

## Abstract

‘Motion dazzle camouflage’ is the name for the putative effects of highly conspicuous, often repetitive or complex, patterns on parameters important in prey capture, such as the perception of speed, direction and identity. Research into motion dazzle camouflage is increasing our understanding of the interactions between visual tracking, the confusion effect and defensive coloration. However, there is a paucity of research into the effects of contrast on motion dazzle camouflage: is maximal contrast a prerequisite for effectiveness? If not, this has important implications for our recognition of the phenotype and understanding of the function and mechanisms of potential motion dazzle camouflage patterns. Here we tested human participants' ability to track one moving target among many identical distractors with surface patterns designed to test the influence of these factors. In line with previous evidence, we found that targets with stripes parallel to the object direction of motion were hardest to track. However, reduction in contrast did not significantly influence this result. This finding may bring into question the utility of current definitions of motion dazzle camouflage, and means that some animal patterns, such as aposematic or mimetic stripes, may have previously unrecognized multiple functions.

## 1. Introduction

A common solution to the need to avoid detection and capture in animals is through camouflage. However, when concealing coloration fails, one theorized mechanism through which coloration may interfere with the capture of an animal is that of so-called motion dazzle camouflage. First suggested by Abbot Thayer [1], motion dazzle camouflage is thought to comprise of high-contrast geometric patterns which may interrupt the systems of motion detection in visual perception and leave the observer unable to correctly perceive the speed or trajectory of the camouflaged object [2,3]. There is support for this hypothesis from studies using human participants [4–7], although results have been variable [8,9]. Recently, a study by Hogan *et al*. [10] indicated that there is a benefit to some high-contrast colorations when targets occur in moving groups of identical animals. The authors suggest that this is evidence that motion dazzle camouflage is interacting with the confusion effect; the phenomenon of decreased predator attack success with increased prey group size (or density [11–13]).

However, few studies of motion dazzle camouflage have explicitly tested the influence of contrast with appropriate controls, although one study by Scott-Samuel *et al*. [5] found distortions in perceived speed for some high-contrast targets moving at fast speeds but not in otherwise identical low-contrast targets. In contrast with this, a study by Stevens *et al*. [7] found that striped targets with low contrast were caught by participants significantly less often than striped targets with high contrast. The relative paucity of research into the influence of contrast on motion dazzle camouflage may be due to the implicit assumption of ‘high contrast’ when discussing motion dazzle camouflage. This would mean that the definition of the pattern includes both function and phenotype. This is a potential route to confusion, particularly when the mechanisms are not well understood [14,15].

The influence of contrast in motion dazzle camouflage is of interest because it is an important aspect it shares with disruptive camouflage [7]. Indeed, the first advocate of both mechanisms of defensive coloration, Thayer [1], discussed both under the same heading of ‘ruptive’ coloration [15]. Disruptive camouflage patterns are described as containing configurations of contours that help to disrupt the form or outline of an animal [16]. These patterns contain high internal contrast, but this ceases to be effective if it results in poor background matching [7,17,18]. There is evidence that disruptive coloration is capable of reducing detection when targets are stationary but not when in motion [19]. Counter to this, it has been found that patterns usually associated with dazzle camouflage (maximally contrasting, i.e. black and white, patterns) appear to come with a cost of increased salience when stationary but benefits when in motion [7]. However, since the necessity for maximal contrast in motion dazzle camouflage is not wholly certain, there remains the possibility that this motion dazzle camouflage may be more common than is previously recognized, due to the restriction of previous research into patterns which have maximal contrast. Indeed the possibility of non-maximally contrasting dazzle camouflage could also mean that this type of defensive coloration may co-occur with other patterns.

The influence of prey aggregation on the effects of motion dazzle camouflage is not well understood, although footage of groups of zebra (*Equus burchelli*) caused more aberrant motion signals in a biologically motivated motion detection model than did footage of single zebra [20]. A recent study by Hughes *et al*. [8] found that striped targets in groups of six were caught more easily by participants than uniform grey targets. By contrast, a study by Hogan *et al*. [10] has since indicated that there are benefits to some striped patterns to targets in larger groups, and where group size remains constant over time. In order to investigate the influence of contrast in dazzle camouflage, participants were asked to play a predator, and track an individual target square among a varying number of distractor squares, with varied coloration patterns and contrast. These included striped patterns, parallel or orthogonal to the direction of motion, plus random black-and-white patterns, all in maximal and lower contrast variants. Although stylized, this approach has been very successful in isolating the mechanism behind many aspects of real-world predator, and anti-predator, behaviour [8,13,17,19,21,22].

## 2. Material and methods

A computer-driven task was created in Matlab (The Mathworks Inc, Natick, MA, USA) using the Psychophysics Toolbox extensions [23–25]. All stimuli were viewed at 62 cm from a gamma-corrected (i.e. linearized) 19^″^ Dell Trinitron CRT monitor (Dell Inc., Round Rock, TX, USA), with a refresh rate of 100 Hz, a resolution of 1024 × 768 pixels, and mean luminance of 71.4 cd m^−2^. At the experimental viewing distance, each pixel subtended 2.2 minarc.

On each trial, subjects were presented with sets of 1, 10, 20, 30, 40, 50 or 60 moving squares which were constrained within a central area on the screen (268 × 268 pixels). Each square was 32 × 32 pixels in size, and moved at 200 pixels s^−1^ (7.54 visual degrees s^−1^). The direction of movement of all squares from one frame to the next can be described as a correlated random walk. The direction of movement of each square in each frame was random, but with weighted probabilities which were described by a circular Gaussian distribution, such that continuing in the same direction was the most probable and more extreme deviations were less probable. The standard deviation of the circular Gaussian distribution was fixed at *π*/8 radians in this experiment, a value that was selected from pilot studies. In the current study, the orientation of the squares matched their direction of movement, such that each square maintained a constant orientation relative to its heading, which allowed the investigation of the effect of oriented colour patterns.

**Figure 1.**
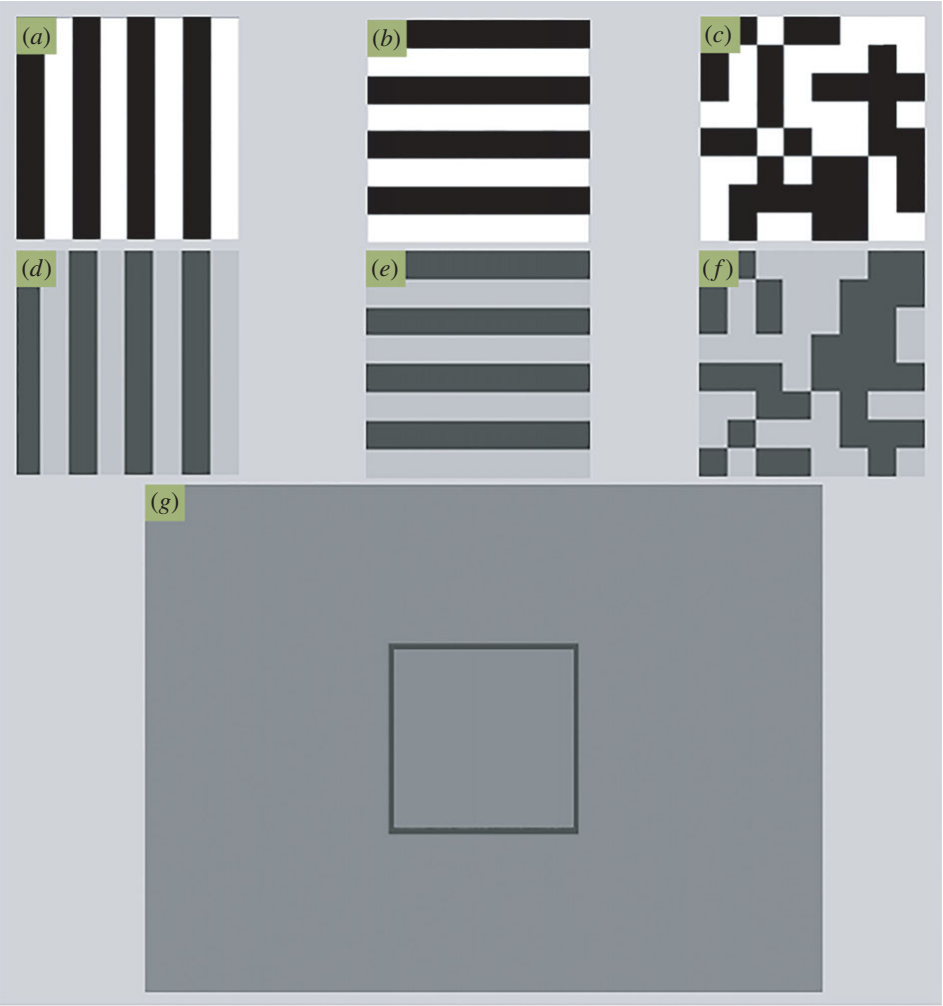
Illustration of the stimuli used: (*a*) Square pattern with stripes orthogonal to horizontal movement, (*b*) square pattern with lines parallel to horizontal movement, (*c*) binary noise square pattern, (*d–f*) lower contrast versions of *a*–*c*, (*g*) example of screen with the mean luminance background.

In each trial, the background upon which the objects were drawn was the mean luminance of the targets (71.4 cd m^−2^, see figure 1*g*). There were three coloration treatments applied to the moving squares, and each of these occurred in either high- or low-contrast conditions. In the high-contrast condition the contrast of the treatment was 100% (0.5 and 132.5 cd m^−2^) and in the lower contrast condition the contrast of the treatment was set at 50% (38 and 103 cd m^−2^). The three coloration treatments applied to the moving squares were either a binary pattern with each 4 × 4 pixel area being either dark or light with equal probability (figure 1*c*), or two coloration patterns each made up of a square-wave grating with wavelength 8 pixels, oriented either parallel or orthogonal in relation to the square's motion (figure 1*a,b*). The phase of the square-wave (starting dark–light or vice versa) was randomly assigned as 0° or 180° with probability 1/2. Each combination of square coloration and contrast were combined factorially to form six conditions.

The participant's task was to track the movements of the target square with a mouse-controlled on-screen cursor (a red circle, so as to provide clear discrimination from the targets and background, with an 8 pixel radius) until the end of a 5000 ms moving period. One of the squares was highlighted for 1000 ms at the onset of each trial, indicating that this was the target square. The Cartesian locations of the centre of the target square and centre of the cursor were recorded every 10 ms. The mean distance of the cursor from the target in pixels for the final 4000 ms of each trial was calculated and recorded. Participants completed four practice trials which were excluded from the analysis, followed by 336 trials in six randomly ordered blocks, one for each combination of coloration condition and contrast level. The order of trials within each block was also randomized independently for each subject. There were 14 participants, who were recruited opportunistically, and each was reimbursed £7 for participation. Each gave their informed written consent in accordance with the Declaration of Helsinki, and the experiment was approved by the Ethical Committee of the Faculty of Science, University of Bristol.

All statistical analysis was performed in R (R Foundation for Statistical Computing, www.R-project.org). Participant mean response errors were distributed approximately lognormally, so were transformed with a natural logarithm for all analyses, which utilized General Linear Mixed Models (function lmer in the lme4 package; [26]). Relaxing the compound symmetry assumption for this repeated measures design, by use of generalized least squares (function gls in package nlme; [27]), produced a very similar result in terms of effect sizes and statistical significance, so we present the simpler analyses here. The most complex model fitted number of distractors as a quadratic polynomial, along with the two factors, target coloration type and contrast level. The first model includes the three-way interaction of these factors, and subsequent models address whether main or interaction effects can instead be modelled as linear terms. The change in deviance between models with and without the predictor variables of interest was tested against a *χ*^2^-distribution with degrees of freedom equal to the difference in degrees of freedom between the models [28].

## 3. Results

**Figure 2.**
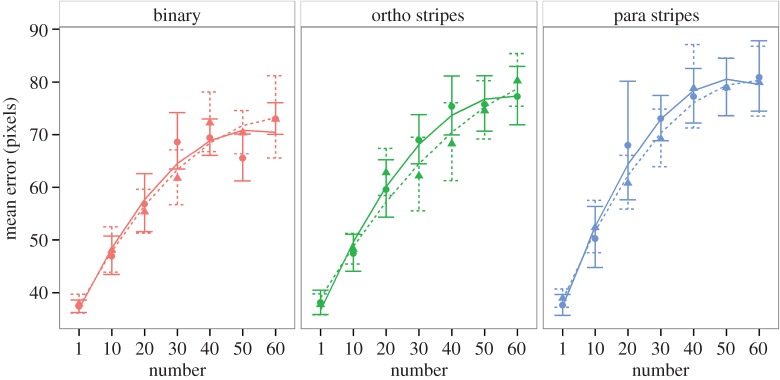
Plots of participant mean tracking error against object number; maximal contrast conditions plotted with solid lines and circular points; lower contrast with dashed lines and triangles. Error bars indicate within-subject 95% confidence intervals.

**Figure 3.**
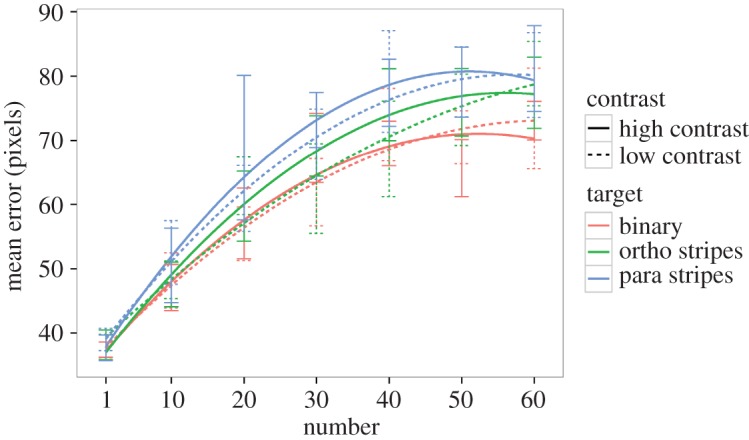
Plot of fitted model, colour indicates target coloration condition and line solidarity indicates contrast condition. Maximal contrast conditions plotted with solid lines. Error bars indicate within-subject 95% confidence intervals.

A model which included all interactions and fitted number as a quadratic polynomial was significantly better than one with a linear fit to number (*χ*^2^ = 198.76, d.f. = 6, *p* < 0.001, AIC −395.33 versus −582.2) so number was fitted as a quadratic polynomial in all following analyses. The three-way interaction between number, contrast and target coloration was not significant (*χ*^2^ = 0.4994, d.f. = 4, *p* = 0.9736, AIC −589.7 versus –582.2). The interaction between target coloration and contrast was not significant (*χ*^2^ = 0.4992, d.f. = 2, *p* = 0.7791, AIC −593.2 versus −589.7) nor was the interaction between contrast and number (*χ*^2^ = 4.4025, d.f. = 2, *p* = 0.1107, AIC −592.79 versus −593.20) or the interaction between target coloration and number (*χ*^2^ = 7.1241, d.f. = 4, *p* = 0.1295, AIC −593.67 versus −592.79; figure 2). However, there were significant main effects of target coloration (*χ*^2^ = 43.14, d.f. = 2, *p* < 0.001, AIC −593.67 versus −554.53) and number (*χ*^2^ = 802.47, d.f. = 2, *p* < 0.001, AIC −593.67 versus 204.80), but no significant main effect of contrast level (*χ*^2^ = 0.62, d.f. = 1, *p* = 0.4303, AIC −593.67 versus −595.53). Post hoc tests on the main effect of target coloration, using Tukey-type control for multiple testing (R package multcomp; [29]), indicate that the parallel striped pattern caused greater errors than both the orthogonally striped one (*z* = 2.464, *p* = 0.0365) and the binary pattern (*z* = 5.064, *p* =  < 0.001), and further that the orthogonally striped pattern caused greater errors than the binary pattern (*z* = 2.599, *p* = 0.0252; figure 3).

## 4. Discussion

The pattern of results found for all coloration and contrast conditions is consistent with the confusion effect; participant tracking errors increased significantly with group size in all cases. There were significant main effects of target coloration condition, with parallel striped patterns causing greater errors than orthogonally striped patterns, which in turn caused greater errors than the binary pattern. In contrast with predictions, there was no significant effect of contrast on tracking errors, at the levels of contrast tested.

Both target coloration conditions with linear contours caused significantly greater errors than the otherwise similar binary condition. This indicates that the presence of linear contours and/or a repetitive pattern is important for the effects of target coloration on tracking, which may support suggestions from the literature that spatio-temporal aliasing and or the aperture problem may be the mechanisms behind motion dazzle camouflage [20]. This result also indicates that patterns which may be high in contrast but do not incorporate regular patterns, for instance typical disruptive camouflage [7,16,18,30] may not maximize benefits for the prey in terms of interference with motion tracking by predators. This is not to say that such patterns have no benefit to moving animals; Hall *et al*. [19] have previously shown that disruptive camouflage patterns impair discrimination between similarly coloured moving targets.

In line with previous work using similar paradigms, tracking errors were greater when targets occurred in larger group sizes. In addition, the effects of target coloration correspond with Hogan *et al*. [10], where parallel striped patterns were found to cause greater tracking errors than orthogonally striped ones or trinary background matching patterns. However in this case, we found no evidence of an increased interaction with the confusion effect (the increase of error with group size) for parallel striped patterns relative to the other patterns. While this could be an artefact of the experimental design, in that since the trinary pattern was not included in the experiment, there is no longer a ‘control’ against which to differ, this may be unlikely because previous findings indicated that the errors caused by orthogonally striped patterns were equivalent to those of background matching patterns. Nevertheless, the overall finding that parallel striped patterns impede target tracking is upheld. This suggests that animals may benefit from such patterns when moving in groups. Phylogenetically controlled correlations of ecology and coloration may corroborate this interpretation; Seehausen *et al*. [31] found that the evolution of longitudinal stripes was correlated with piscivorous feeding modes and with shoaling behaviour, ecological parameters which may suggest a need for camouflage that works in moving groups of animals. Additionally, Allen *et al*. [32] found that snakes with longitudinal stripes were generally fast, small and often exposed to visual hunters, indicating that the animal may be under selection for coloration that is effective in movement masking.

Contrary to the results of Scott-Samuel *et al*. [5] who found an advantage to high contrast, and the results of Stevens *et al*. [7] who found an advantage to low contrast, we found no significant effect of contrast. These differences could stem from the experimental designs used; Scott-Samuel *et al.*'s experiment interrogated speed judgements, and Stevens *et al.*'s target capture, whereas here we measure target tracking. Alternatively, the levels of contrast used may account for the differences; Scott-Samuel *et al*. used far lower levels in their low-contrast treatment (6.25% compared to our 50%), although Stevens *et al*. used levels of contrast more similar to those used here. Future research should try a range of contrast levels on a range of backgrounds, to ascertain the relationship between contrast and motion dazzle effects. If upheld, as it is for moving patterns (dynamic dazzle) [33] the lack of the necessity for maximal contrast may also have wider implications for the understanding of which patterns may constitute motion dazzle camouflage. The commonly cited definitions of motion dazzle camouflage include both function and phenotype, but if there are patterns that have the same function (and mechanisms) but mismatching phenotype the utility of the current definitions may become questionable.

If maximal contrast is not necessary to invoke the mechanisms of dazzle camouflage, this could have wide-ranging implications for the understanding of motion dazzle coloration. It has been demonstrated that high-contrast patterns may have advantages for moving animals but disadvantages for stationary animals [7]. However, if maximal contrast is unnecessary, it may be the case that animal patterns only have to maintain a certain level of contrast for the animal to benefit from motion dazzle when moving. This could minimize the costs of dazzle camouflage, and make the possibility of dual function or compromise patterns including dazzle camouflage more realistic, indeed many animal patterns not considered to constitute motion dazzle contain repetitive elements, most obviously the aposematic patterns of wasps, bees and their hoverfly mimics [34–36]. This would also considerably widen the pool of possible instances of motion dazzle camouflage by moving the focus away from maximally contrasting (black and white) patterns into any repetitive patterns with a threshold level of contrast.

## Supplementary Material

Experimental Dataset; a .txt file containing data from all trials for all participants

## Data accessibility

The datasets supporting this article have been uploaded as part of the electronic supplementary material.

## References

[1] Thayer GH 1909 Concealing-coloration in the animal kingdom: an exposition of the laws of disguise through color and pattern: being a summary of Abbott H. Thayer's discoveries. New York, NY: Macmillan.

[2] Behrens RR 1999 The role of artists in ship camouflage during World War I. Leonardo 32, 53–59. (doi:10.1162/002409499553000)

[3] Williams DL 2001 Naval Camouflage 1914–1945: a complete visual reference. London, UK: Chatham.

[4] Hughes AE, Troscianko J, Stevens M 2014 Motion dazzle and the effects of target patterning on capture success. BMC Evol. Biol. 14, 201 (doi:10.1186/s12862-014-0201-4) 25213150 10.1186/s12862-014-0201-4 PMC4172783

[5] Scott-Samuel NE, Baddeley R, Palmer CE, Cuthill IC 2011 Dazzle camouflage affects speed perception. PLoS ONE 6, e20233 (doi:10.1371/journal.pone.0020233) 21673797 10.1371/journal.pone.0020233 PMC3105982

[6] Stevens M, Yule DH, Ruxton GD 2008 Dazzle coloration and prey movement. Proc. R. Soc. B 275, 2639–2643. (doi:10.1098/rspb.2008.0877) 10.1098/rspb.2008.0877 PMC2605810 18700203

[7] Stevens M, Searle WTL, Seymour JE, Marshall KL, Ruxton GD 2011 Motion dazzle and camouflage as distinct anti-predator defenses. BMC Biol. 9, 81 (doi:10.1186/1741-7007-9-81) 22117898 10.1186/1741-7007-9-81 PMC3257203

[8] Hughes AE, Magor-Elliott RS, Stevens M 2015 The role of stripe orientation in target capture success. Front. Zool. 12, 17 (doi:10.1186/s12983-015-0110-4) 26269704 10.1186/s12983-015-0110-4 PMC4533824

[9] von Helversen B, Schooler LJ, Czienskowski U 2013 Are stripes beneficial? Dazzle camouflage influences perceived speed and hit rates. PLoS ONE 8, e61173 (doi:10.1371/journal.pone.0061173) 23637795 10.1371/journal.pone.0061173 PMC3634837

[10] Hogan BG, Cuthill IC, Scott-Samuel NE 2016 Dazzle camouflage, target tracking and the confusion effect. Behav. Ecol. 27 (doi:10.1093/beheco/arw081) 10.1093/beheco/arw081 PMC5027625 27656087

[11] Krakauer DC 1995 Groups confuse predators by exploiting perceptual bottlenecks: a connectionist model of the confusion effect. Behav. Ecol. Sociobiol. 36, 421–429. (doi:10.1007/BF00177338)

[12] Landeau L, Terborgh J 1986 Oddity and the ‘confusion effect’ in predation. Anim. Behav. 34, 1372–1380. (doi:10.1016/S0003-3472(86)80208-1)

[13] Scott-Samuel NE, Holmes G, Baddeley R, Cuthill IC 2015 Moving in groups: how density and unpredictable motion affect predation risk. Behav. Ecol. Sociobiol. 69, 867–872. (doi:10.1007/s00265-015-1885-1) 25983380 10.1007/s00265-015-1885-1 PMC4425808

[14] Ruxton GD, Speed MP, Kelly DJ 2004 What, if anything, is the adaptive function of countershading? Anim. Behav. 68, 445–451. (doi:10.1016/j.anbehav.2003.12.009)

[15] Stevens M, Merilaita S 2009 Defining disruptive coloration and distinguishing its functions. Phil. Trans. R. Soc. B 364, 481–488. (doi:10.1098/rstb.2008.0216) 18990673 10.1098/rstb.2008.0216 PMC2674077

[16] Cuthill IC, Stevens M, Sheppard J, Maddocks T, Párraga CA, Troscianko TS 2005 Disruptive coloration and background pattern matching. Nature 434, 72–74. (doi:10.1038/nature03312) 15744301 10.1038/nature03312

[17] Fraser S, Callahan A, Klassen D, Sherratt TN 2007 Empirical tests of the role of disruptive coloration in reducing detectability. Proc. R. Soc. B 274, 1325–1331. (doi:10.1098/rspb.2007.0153) 10.1098/rspb.2007.0153 PMC2176178 17360282

[18] Stevens M, Cuthill IC, Windsor AMM, Walker HJ 2006 Disruptive contrast in animal camouflage. Proc. R. Soc. B 273, 2433–2438. (doi:10.1098/rspb.2006.3614) 10.1098/rspb.2006.3614 PMC1634902 16959632

[19] Hall JR, Cuthill IC, Baddeley R, Shohet AJ, Scott-Samuel NE 2013 Camouflage, detection and identification of moving targets. Proc. R. Soc. B 280, 20130064 (doi:10.1098/rspb.2013.0064) 10.1098/rspb.2013.0064 PMC3619462 23486439

[20] How MJ, Zanker JM 2014 Motion camouflage induced by zebra stripes. Zool. Jena Ger. 117, 163–170. (doi:10.1016/j.zool.2013.10.004) 10.1016/j.zool.2013.10.004 24368147

[21] Ruxton GD, Jackson AL, Tosh CR 2007 Confusion of predators does not rely on specialist coordinated behavior. Behav. Ecol. 18, 590–596. (doi:10.1093/beheco/arm009)

[22] Sherratt TN, Rashed A, Beatty CD 2004 The evolution of locomotory behavior in profitable and unprofitable simulated prey. Oecologia 138, 143–150. (doi:10.1007/s00442-003-1411-4) 14564501 10.1007/s00442-003-1411-4

[23] Brainard DH 1997 The psychophysics toolbox. Spat. Vis. 10, 433–436. (doi:10.1163/156856897X00357) 9176952

[24] Kleiner M, Brainard DH, Pelli GD 2007 What's new in Psychtoolbox-3? *Perception* **36**, S14.

[25] Pelli GD 1997 The VideoToolbox software for visual psychophysics: transforming numbers into movies. Spat. Vis. 10, 437–442. (doi:10.1163/156856897X00366) 9176953

[26] Bates D, Maechler M, Bolker B, Walker S 2015 Fitting linear mixed-effects models using Ime4. J. Stat. Softw. 67, 1–48. (doi:10.18637/jss.v067.i01)

[27] Pinheiro J, Bates D, DebRoy S, Sarkar D, R-core team 2016 nlme: Linear and nonlinear mixed effects models. R package version 3.1-128. See http://CRAN.R-project.org/package=nlme.

[28] Crawley MJ 2007 The R Book. Hoboken, NJ: Wiley-Blackwell.

[29] Hothorn T, Bretz F, Westfall P 2008 Simultaneous inference in general parametric models. Biom. J. Biom. Z. 50, 346–363. (doi:10.1002/bimj.200810425) 10.1002/bimj.200810425 18481363

[30] Merilaita S 1998 Crypsis through disruptive coloration in an isopod. Proc. R. Soc. Lond. B 265, 1059–1064. (doi:10.1098/rspb.1998.0399)

[31] Seehausen M, Alphen JJM 1999 Evolution of colour patterns in East African cichlid fish. J. Evol. Biol. 12, 514–534. (doi:10.1046/j.1420-9101.1999.00055.x)

[32] Allen WL, Baddeley R, Scott-Samuel NE, Cuthill IC 2013 The evolution and function of pattern diversity in snakes. Behav. Ecol. 24, 1237–1250. (doi:10.1093/beheco/art058)

[33] Hall JR, Cuthill IC, Baddeley R, Attwood AS, Munafò MR, Scott-Samuel NE 2016 Dynamic dazzle distorts speed perception. PLoS ONE 11, e0155162 (doi:10.1371/journal.pone.0155162) 27196098 10.1371/journal.pone.0155162 PMC4872993

[34] Sherratt TN 2002 The evolution of imperfect mimicry. Behav. Ecol. 13, 821–826. (doi:10.1093/beheco/13.6.821)

[35] Speed MP, Ruxton GD 2010 Imperfect Batesian mimicry and the conspicuousness costs of mimetic resemblance. Am. Nat. 176, E1–E14. (doi:10.1086/652990) 20497052 10.1086/652990

[36] Stevens M, Ruxton GD 2012 Linking the evolution and form of warning coloration in nature. Proc. R. Soc. B 279, 417–426. (doi:10.1098/rspb.2011.1932) 10.1098/rspb.2011.1932 PMC3234570 22113031

